# Epilepsy, impaired functioning, and quality of life in patients with tuberous sclerosis complex

**DOI:** 10.1002/epi4.12365

**Published:** 2019-10-27

**Authors:** Menno Vergeer, Wendela L. de Ranitz‐Greven, Maureen P. Neary, Raluca Ionescu‐Ittu, Bruno Emond, Mei Sheng Duh, Floor Jansen, Bernard A. Zonnenberg

**Affiliations:** ^1^ University Medical Center Utrecht Utrecht Netherlands; ^2^ Novartis Pharmaceuticals Corporation East Hanover NJ USA; ^3^ Analysis Group, Inc. Montréal Québec Canada; ^4^ Analysis Group, Inc. Boston MA USA

**Keywords:** epilepsy, outcome research, quality of life, seizures

## Abstract

**Objective:**

To estimate health‐related quality of life (HRQoL) in patients with tuberous sclerosis complex (TSC) and associated manifestations and to identify potential factors associated with HRQoL in this population of patients.

**Methods:**

We performed a retrospective chart review of adults with TSC who attended the outpatient clinic of the University Medical Center Utrecht in the Netherlands from 1990 to 2015 (N = 363; on average 33.6 years of follow‐up). HRQoL data were assessed in 2012 using the Health Utility Index version 3 (HUI‐3) questionnaire completed by patients or caregivers (N = 214 with HUI score and ≥1 TSC manifestation, including renal angiomyolipomas [rAMLs], subependymal giant cell astrocytoma [SEGA], or epilepsy).

**Results:**

Of 214 patients in the study sample, 171 had TSC‐associated epilepsy (with or without rAML/SEGA), 37 had TSC and rAML (without epilepsy or SEGA), and 6 had other combinations of manifestations. The median HUI score for the 214 patients with ≥1 TSC manifestation was 0.51 (−0.371 to 1 scale, 1 = perfect health, 0 = death, <0 = worse than death). Among all components used to build the overall HUI score, the cognition component had the lowest score (mean = 0.47; 0‐1 scale). Patients with TSC‐epilepsy had significantly lower overall HUI than patients with TSC and rAML only (median HUI = 0.31 vs 0.95, *P* < .05), especially those who were in refractory state for prolonged periods of time (median HUI = −0.11 among patients with seizures during the entire duration of their follow‐up time). In multivariate analyses, severe impairment of daily functioning was the strongest predictor of HRQoL decrement (adjusted HUI difference between patients with severe vs. no impairment = −0.55, *P* < .05).

**Significance:**

This study showed that TSC‐related epilepsy is associated with lower HUI, especially for patients who have refractory seizures for prolonged periods of time. Early and effective interventions to control or reduce seizures and preserve patients’ cognitive functions may help to improve patients’ quality of life.

**Key points**

- The objective was to estimate health‐related quality of life (HRQoL) in patients with tuberous sclerosis complex (TSC) and identify factors predicting HRQoL.
- The Health Utility Index version 3 (HUI‐3) was lowest among patients with TSC‐associated epilepsy compared to patients with TSC who did not have epilepsy.
- Among patients with TSC‐associated epilepsy, those with refractory epilepsy had lower HUI versus those without.
- Severe impairment of daily functioning was the strongest predictor of decrements in HUI.
- The results from this study support the necessity of early and effective treatment interventions to reduce or control seizures.

## 1 INTRODUCTION

Tuberous sclerosis complex (TSC) is an autosomal dominant disease characterized by the growth of benign tumors in multiple organ systems. Renal angiomyolipomas (rAMLs), subependymal giant cell astrocytoma (SEGA), and epilepsy are manifestations of TSC that pose a significant burden on patients.^1^, ^2^ rAMLs occur in 23%‐69% of patients with TSC^3^, ^4^, ^5^, ^6^ and can lead to potentially life‐threatening hemorrhage due to ruptured aneurysms, and impaired renal function.^7^ SEGAs are relatively slow‐growing brain tumors,^8^, ^9^ which occur in approximately 5%‐20% of individuals with TSC.^10^, ^11^, ^12^, ^13^, ^14^ Epilepsy is one of the most common manifestations of TSC, with a prevalence of 83.5%, as reported in a large TSC registry.^15^ In 67% of cases, the onset of TSC‐associated epilepsy occurs in the first two years of life and 62.5% may develop refractory epilepsy.^16^, ^17^ In patients with refractory epilepsy, the prevalence of impaired daily functioning is particularly high.^18^ Patients with TSC‐associated epilepsy have also been reported to have poor health‐related quality of life (HRQoL)^19^, ^20^, ^21^ particularly those with refractory seizures who may develop nonreversible neuropsychiatric problems and cognitive deficits.^21^ Conversely, low seizure frequency is associated with significantly higher HRQoL.^22^

Data on the impact of these three TSC‐related manifestations on the HRQoL of patients with TSC are still scarce. The main objectives of this study were to: (a) evaluate HRQoL in patients with different manifestations of TSC with a focus on epilepsy, rAML, and SEGA and (b) identify potential factors associated with HRQoL in patients with TSC. As a secondary objective, this study aimed to describe two subgroups of patients hypothesized a priori to have high disease burden: patients with TSC and refractory epilepsy and patients with TSC and impairment of daily functioning.

## 2 METHODS

### 2.1 Study design and patient selection

This single‐center retrospective chart review study included patients with a definite diagnosis of TSC (based on the Revised 1998 Criteria^23^) and associated manifestations, mainly rAML, SEGA, and epilepsy. All patients attended the outpatient clinic of the University Medical Center of Utrecht (UMCU) between 1990 and 2015, a center of excellence for patients with TSC in The Netherlands. All patients were seen, tested, diagnosed, and treated as part of routine clinical care at UMCU. Patients were followed for an average of 33.6 years.

Most patients with TSC in the study sample had rAML, as assessed by routine screening ultrasound or a computerized tomography (CT) scan. Some patients with rAML were referred for a second opinion or presented at the UMCU Emergency Department with acute renal bleeding. Information on patient TSC and rAML characteristics was extracted from patients’ charts at the UMCU. Additional data on epilepsy (eg, type of seizures, seizure frequency, refractory epilepsy status, use of antiepileptic drugs [AEDs], epilepsy surgery, and vagal nerve stimulation [VNS]), response to treatment, magnetic resonance imaging (MRI), and CT scan were collected from either patients’ UMCU charts or alternatively from available external sources (ie, records from pharmacies from which patients received their AED treatments and neurologists/epileptologists in other medical centers, who shared the care for these patients).

### 2.2 Standard protocol approvals, registrations, and patient consents

The study was approved by the UMCU institutional review board (study METC 14/412C) and patients consented to participate.

### 2.3 HRQoL outcomes and measurements

HRQoL was measured using a generic utilities scale, the Health Utility Index version 3 (HUI‐3).^24^ The HUI questionnaire was sent in 2012 by mail to all patients with TSC who were managed at the UMCU. A patient version and a caregiver version of the questionnaire were provided. The HUI‐3 is a validated “Multi‐Attribute Health Status Classification System” that consists of 8 attributes including vision, hearing, speech, ambulation/mobility, pain, dexterity, emotion, and cognition. HUI‐3 can be used to potentially identify up to 972 000 unique health states. For each health dimension, the respondent must choose between five or six responses, with the first response option corresponding to the best health status (eg, for cognition dimension: “*Able to remember most things, think clearly and solve day to day problems*”) and the last response option corresponding to worst health (eg, for cognition dimension: “*Unable to remember anything at all, and unable to think or solve day‐to‐day problems*”). Each of the eight HUI‐3 dimensions is assigned a score on a 0 to 1 scale, with 0 corresponding to the worst health state for that dimension and 1 corresponding to the best health status for that dimension. The eight dimension‐specific scores can then be combined into an overall HUI score (−0.371 to 1 scale, where 0 corresponds to death, 1 to perfect health, and negative scores represent health states considered “worse than dead”). The overall HUI score is computed using the following formula: *HUI‐3 score = (1.371 × vision ×hearing × speech × ambulation/mobility × pain ×dexterity × emotion ×cognition) – 0.371.* Differences of ≥0.03 in the overall HUI‐3 score are considered to be clinically important.^24^ Referenced Global HUI scores in different populations are presented in Table S1.^25^, ^26^, ^27^, ^28^, ^29^, ^30^, ^31^, ^32^, ^33^

Among patients with TSC–epilepsy, periods with refractory epilepsy were derived from AED treatment response information recorded in the patient's chart at each visit over the follow‐up time (types of AED responses recorded: seizure‐free, 50%‐99% reduction in seizure frequency, <50% reduction in seizure frequency, no response, and worsening). Patients were classified as having refractory epilepsy during the period when they were not seizure‐free while on AED treatment or the periods when the physician reported refractory epilepsy directly in the patient's chart.

The group of patients having TSC and rAML was divided into two subgroups based the size of the largest rAML at the time of the first rAML assessment at the UMCU: patients with large rAMLs (≥3.5 cm) and patients with small rAMLs (<3.5 cm). The 3.5 cm cutoffs for the size of rAMLs was routinely used in the UMCU practice to classify patients and corresponds to the cutoffs between stages two and three in the rAML staging criteria.^34^, ^35^

Impairment of daily functioning was recorded in the patients’ charts as severe (completely dependent in daily life activities), mild to moderate (needing assistance in daily life activities), or no impairment (independent) based on physician assessment.

### 2.4 Statistical analyses

**Figure 1:**
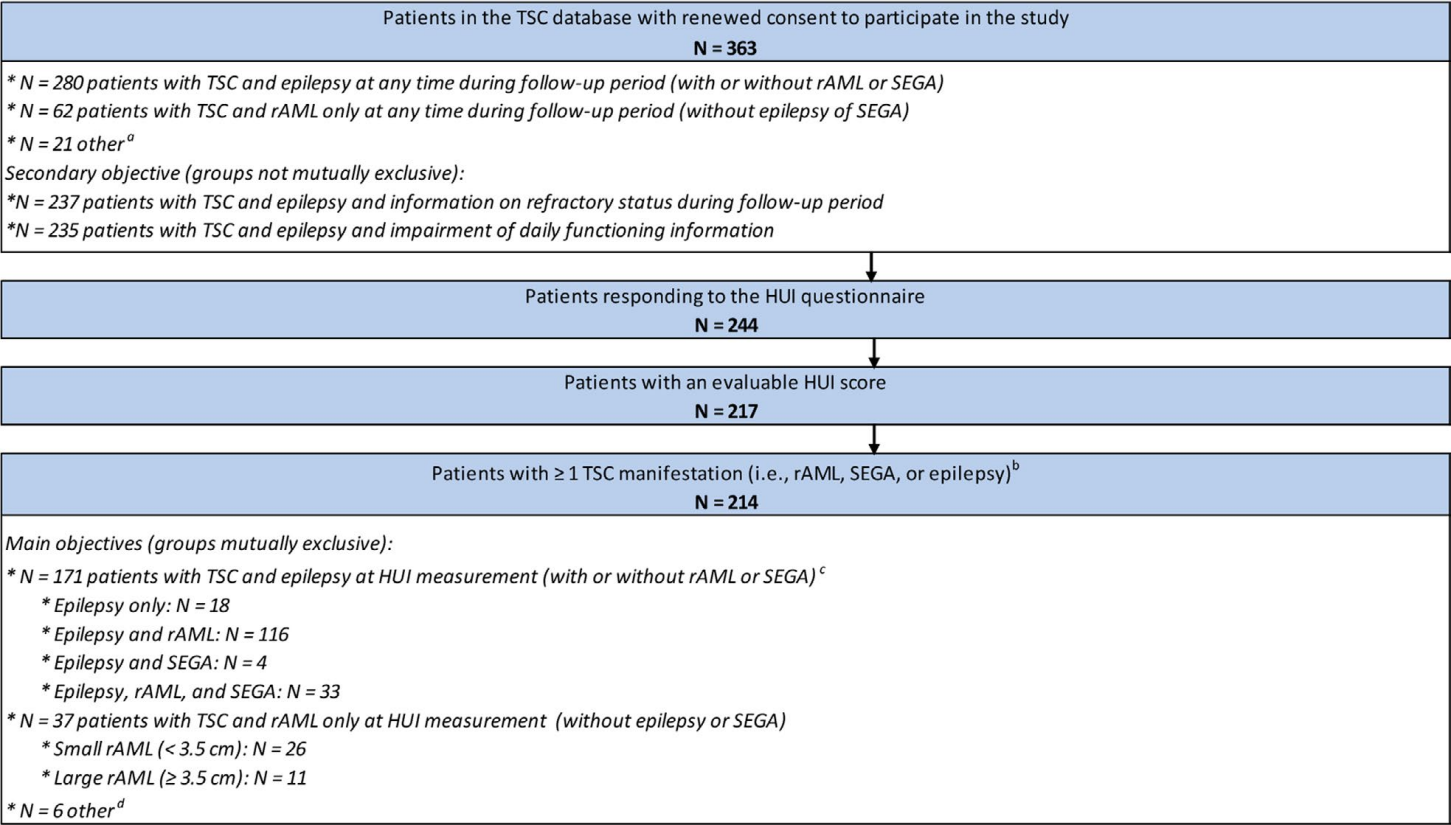
Patient disposition. HUI, Health Utility Index; rAML, renal angiomyolipoma; SEGA, subependymal giant cell astrocytoma; TSC, tuberous sclerosis complex. ^a^Patients with TSC‐rAML‐SEGA without epilepsy, SEGA only, and patients with no active TSC manifestations at the time of the study. ^b^Only rAML, SEGA, and epilepsy manifestations were considered for this analysis. ^c^All patients with TSC and epilepsy were grouped in a single category for the descriptive statistics on HRQoL because preliminary analyses indicated minimal differences in HRQoL between the different subgroups of patients with TSC and epilepsy. ^d^Patients with TSC‐rAML‐SEGA without epilepsy (none of the 214 patients had TSC and SEGA without rAML or epilepsy manifestations)

The HRQoL analyses (main study objectives) were conducted among patients having a valid HUI score and ≥1 TSC manifestation (N = 214; Figure 1). The overall HUI score (HUI distribution, including minimum, first quartile, median and mean, third quartile, and maximum; −0.371 to 1 scale) as well as the scores for each health dimension (component means; 0‐1 scale) were described separately in the full TSC sample and in subgroups of patients with different TSC manifestations. Factors potentially associated with HRQoL in patients with TSC were examined using univariate and multivariate linear regression models from which unadjusted and adjusted overall HUI differences and corresponding 95% confidence intervals (CIs) were reported. The regression models included the following potential predictors identified a priori based on prior literature and clinical input: demographic factors (age, gender), TSC‐specific factors (*TSC2* mutation), presence of TSC manifestations (rAML, SEGA, and epilepsy), and epilepsy‐related factors (epilepsy‐related events and refractory status). More specifically, for epilepsy‐related factors, patients were divided into four mutually exclusive subgroups: (a) patients with refractory epilepsy and a history of epilepsy‐related events, that is, bilateral tonic‐clonic status epilepticus, nonconvulsive status epilepticus, focal status epilepticus, other status epilepticus, unspecified status epilepticus, fractures, injury, intoxication, wounds, behavioral impairment, cognitive decline, and neurological insult; (b) patients with refractory epilepsy without a history of epilepsy‐related events; (c) patients without refractory epilepsy and a history of epilepsy‐related events; and (d) patients without refractory epilepsy and without a history of epilepsy‐related events (used as the reference group “no epilepsy” in regression models).

For the secondary objective, patient characteristics were compared between subgroups of patients stratified by period of time with refractory seizures (ie, proportion of follow‐up time during which the patient was not seizure‐free) and by impairment of daily functioning. For the former stratification, four mutually exclusive subgroups were created among all patients with TSC–epilepsy and information on refractory status (N = 237; Figure 1): patients who were refractory to AEDs 100%, 50%‐99.9%, 0.1%‐50%, or 0% of their follow‐up time. For the latter stratification, two subgroups were created among all patients with TSC and information on impairment of daily functioning (N = 235; Figure 1): patients with impairment of daily functioning (ie, mild, moderate, or severe impairment) and patients without impairment of daily functioning. Patient characteristics were compared across subgroups, using chi‐square test for categorical variables and the *t* test for continuous variables.

## 3 RESULTS

### 3.1 Study sample

Of 363 adult patients with TSC followed at the UMCU between 1990 and 2015, 280 patients had TSC‐associated epilepsy (with or without rAML/SEGA), 62 had TSC and rAML (without epilepsy or SEGA), and 21 had other combinations of TSC manifestations or no TSC manifestations. Among those patients with TSC‐associated epilepsy, 237 had information on refractory status during the follow‐up and 235 had information on impairment of daily functioning.

After restricting the analyses to patients who completed the HUI questionnaire (67% response rate), had a valid HUI score, and had ≥1 TSC manifestation, the study sample for the HUI analyses (N = 214) included 171 patients with TSC‐associated epilepsy (with or without rAML/SEGA), 37 with TSC and rAML (without epilepsy or SEGA), and 6 with TSC‐rAML‐SEGA without epilepsy (results were not reported for this subgroup of patients due to small sample size). None of the 214 patients had TSC and SEGA without rAML or epilepsy manifestations (Figure 1). Among those with TSC‐associated epilepsy, 145 had information on refractory status during the follow‐up.

### 3.2 Main objective 1—HUI in patients with TSC and different manifestations

**Figure 2:**
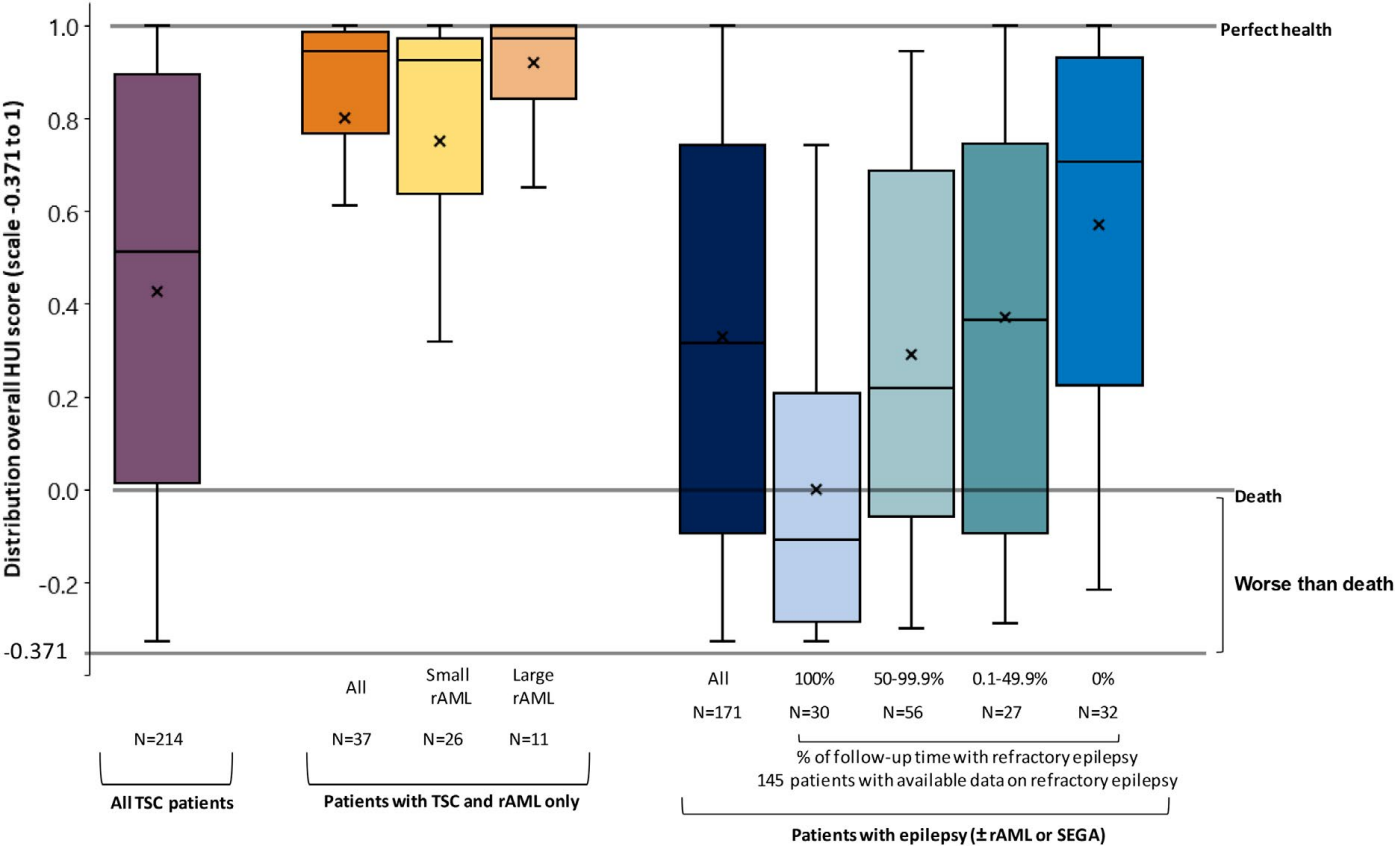
Distribution of overall HUI, by the overall study sample and by study cohorts^a^. HUI, Health Utility Index; rAML, renal angiomyolipoma; SEGA, subependymal giant cell astrocytoma; TSC, tuberous sclerosis complex. ^a^Box plots illustrate, from bottom up: 1.5 times less than first quartile, first quartile, median, third quartile and 1.5 times more than the third quartile value in the distribution. "x" shows the mean of the distribution

Overall HUI scores were analyzed in the complete study sample (N = 214) and in several subgroups (Figure 2). The median overall HUI score for all patients with TSC was 0.51 (interquartile range: 0.01‐0.89; mean: 0.43; on a −0.371 to 1 scale). Patients with TSC and rAML only (N = 37) had significantly higher mean HUI scores than patients with TSC who had epilepsy (N = 171; median HUI: 0.95 vs 0.31; mean HUI: 0.80 vs 0.33, *P* < .05). Among patients with TSC‐rAML only, no statistically significant difference in mean HUI score was observed between patients with small and large rAMLs (median HUI: 0.93 vs 0.97; mean HUI: 0.75 vs 0.92, *P* = .15). Larger differences in HUI scores were observed in patients with TSC‐associated epilepsy and available information on refractory status (N = 145) depending on the period of time with refractory epilepsy (median HUI for patients who were refractory to AEDs 100% vs 0% of follow‐up time: −0.11 vs 0.71; mean HUI: 0.00 vs 0.57, *P* < .05).

**Figure 3:**
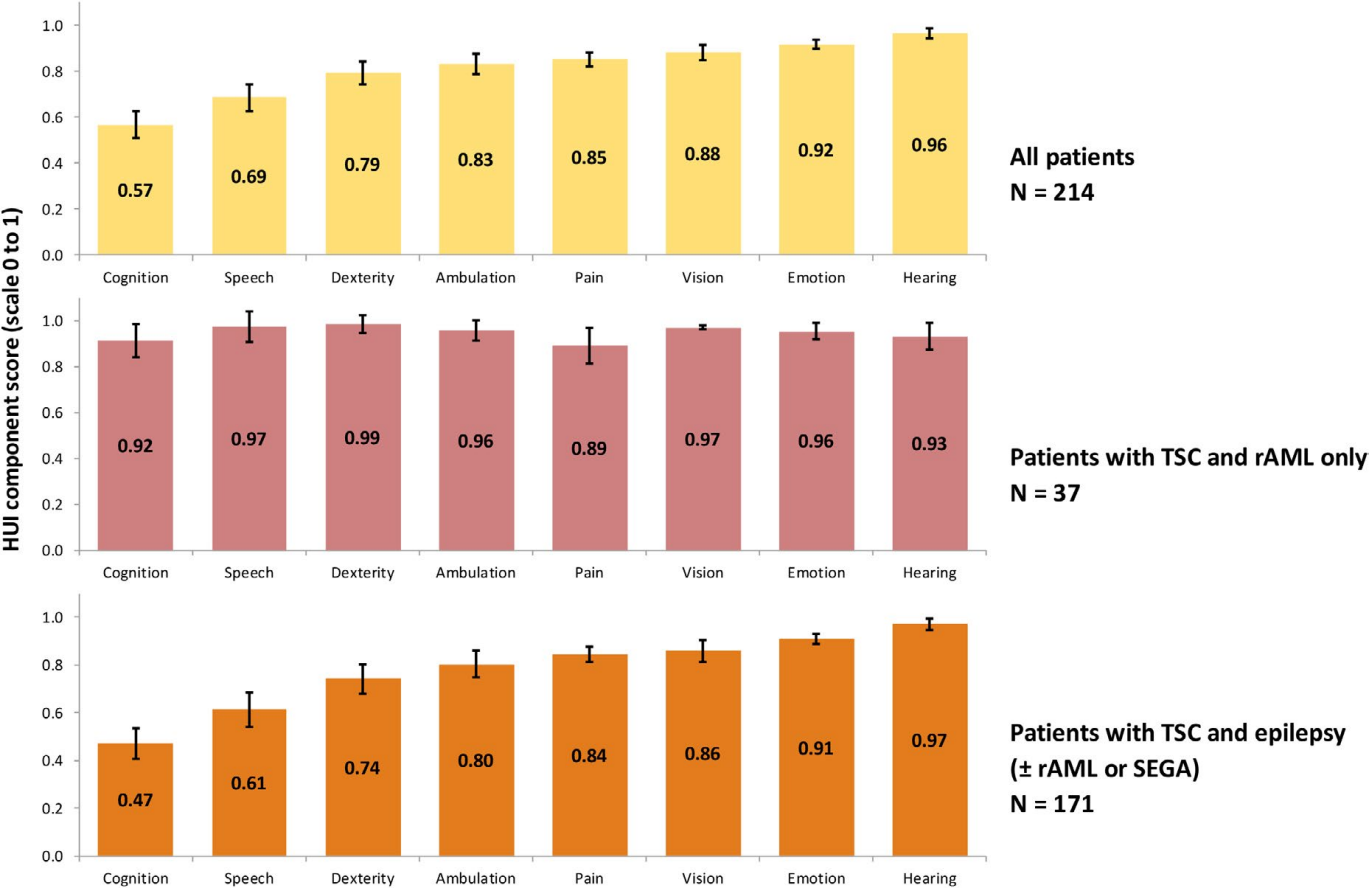
Dimension‐specific HUI, by the overall study sample and by study cohorts. HUI, Health Utility Index; rAML, renal angiomyolipoma; SEGA, subependymal giant cell astrocytoma; TSC, tuberous sclerosis complex

The dimension‐specific scores (Figure 3) showed that the low overall HUI in the complete study sample (N = 214) and among patients with TSC and epilepsy (N = 171) was driven primarily by the cognition dimension (mean HUI in patients with TSC and epilepsy: 0.47; on a 0‐1 scale), and, to a lesser extent, by the speech, dexterity, and ambulation/mobility dimensions. Among patients with TSC‐rAML only, the HUI components with the lowest scores (but considerably higher than in the other patient groups) were cognition and pain (0.92 and 0.89, respectively; on a 0‐1 scale).

### 3.3 Main objective 2—Variables associated with HUI in patients with TSC and different manifestations

**Figure 4:**
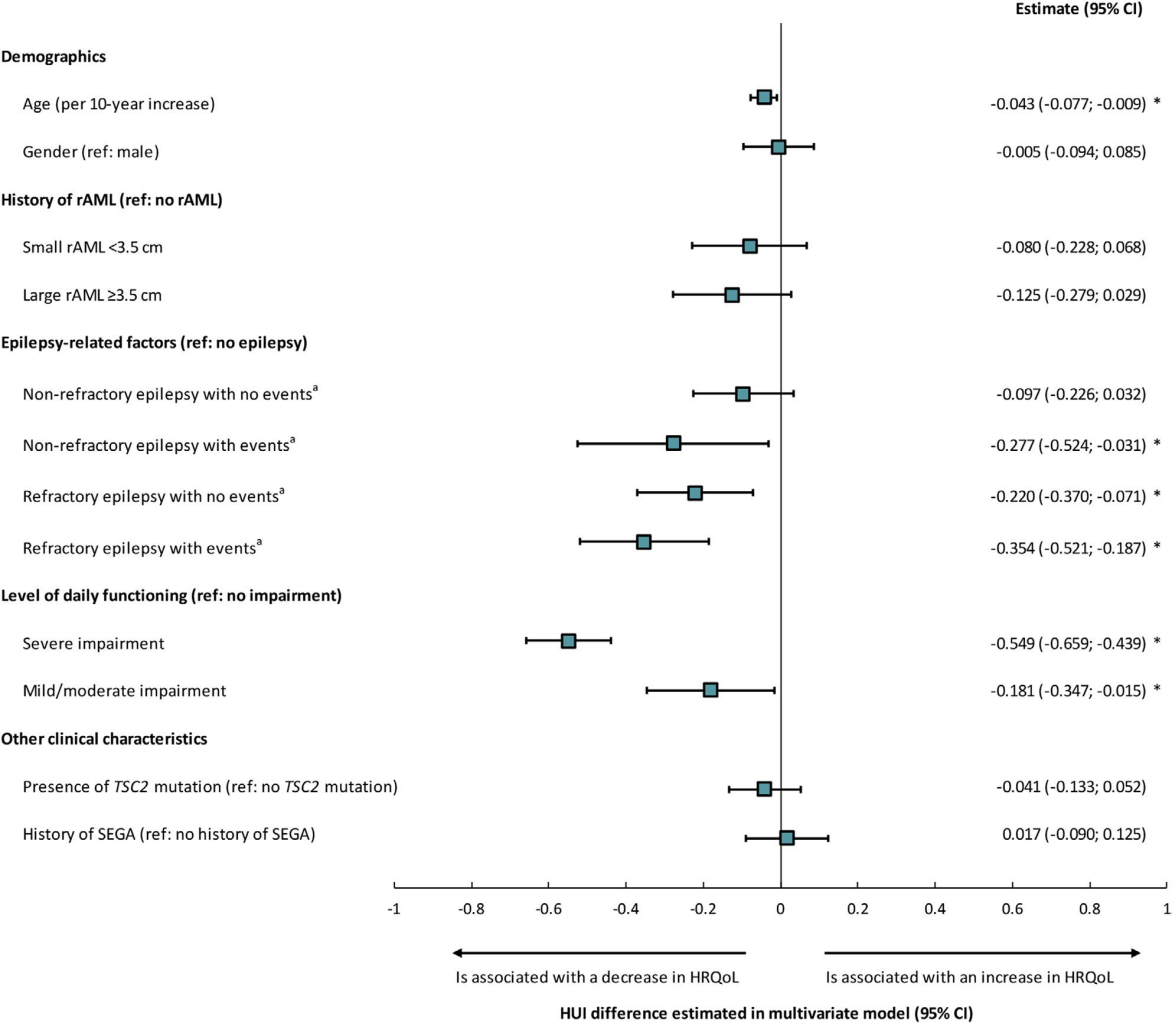
Factors associated with HRQoL score in patients with TSC—multivariate model. *Indicates *P*‐value < .05. *Note:* ^a^Events include bilateral tonic‐clonic status epilepticus, nonconvulsive status epilepticus, focal status epilepticus, other status epilepticus, unspecified status epilepticus, fractures, injury, poisoning, wounds, behavioral worsening, cognitive decline, and neurological insult. AED, antiepileptic drug; CI, confidence interval; rAML, renal angiomyolipoma; HRQoL, health‐related quality of life; HUI, Health Utility Index; SEGA, subependymal giant cell astrocytoma; TSC, tuberous sclerosis complex

Of the factors examined to determine their association with HUI scores in patients with TSC, the following were found to be significantly (*P* < .05) associated with lower HUI scores: older age, refractory epilepsy with or without epilepsy‐related events, nonrefractory epilepsy with epilepsy‐related events, and severe or mild to moderate impairment of daily functioning (Figure 4). Among these, severe impairment of daily functioning had the strongest association with lower HRQoL (HUI difference vs. no impairment of daily functioning: −0.55, *P* < .05), followed by refractory epilepsy with epilepsy‐related events (HUI difference vs. no epilepsy: −0.35, *P* < .05). All associations were stronger in univariate analyses (Figure S1).

### 3.4 Secondary objective—Characteristics of patients stratified by period of time with refractory seizures and by impairment of daily functioning

Description and comparison of patients with TSC‐associated epilepsy (N = 237) stratified by the proportion of follow‐up time spent in a refractory state (ie, not seizure‐free) are provided in Table 1. In all subgroups, epilepsy information was collected over a median follow‐up time ranging from 28 to 35 years. The time spent in a refractory state was strongly associated with patients’ HRQoL, both in terms of overall HUI (patients with 100% vs 0% of follow‐up time with refractory epilepsy: overall HUI median −0.11 vs 0.71, respectively, on a −0.371 to 1 scale) and cognitive HUI (median 0 vs 0.86, respectively, on a 0‐1 scale). The distribution of gender was not different between the four subgroups, but patients who were seizure‐free over the full follow‐up were somewhat older than the patients in the other subgroups (median age: 53 vs 42‐46 years). Patients who were in a refractory state for a longer period of time were more likely to have seizures with bilateral motor symptoms, to have a *TSC2* mutation, to live in a group home or with a caregiver, to have severe impairment of daily functioning, to use a higher number of AEDs, and to have a history of epilepsy‐related events, particularly bilateral tonic‐clonic status epilepticus and fractures. Healthcare resource utilization was also higher in patients who were in a refractory state for a longer period of time, with the exception of those who were in a refractory state 100% of the time (of note, this group includes patients who are institutionalized, and may receive their medical care directly in this setting).

**Table 1:** Characteristics of patients with TSC–epilepsy stratified by the proportion of follow‐up time in refractory state

	Patients with TSC–epilepsy having data on refractory epilepsy (N = 237)			
	Proportion of follow‐up time in refractory state (based on refractory flag and treatment response in the data)			
	100.0%	50.0%‐99.9%	0.1%‐50.0%	0.0%
	N = 49	N = 81	N = 44	N = 63
Follow‐up, y, mean ± SD [median]	30.7 ± 9.7 [27.7]	36.4 ± 10.3 [34.7]*	36.0 ± 19.7 [31.5]	30.4 ± 17.6 [27.7]
Time with refractory epilepsy, y, mean ± SD [median]	30.7 ± 9.7 [27.7]	28.3 ± 8.9 [27.7]	8.7 ± 8.2 [6.4]*	0.0 ± 0.0 [0.0]*
Quality of life				
HUI measurement, n (%)	30 (61.2%)	56 (69.1%)	27 (61.4%)	32 (50.8%)
HUI score, mean ± SD [median]	0.00 ± 0.32 [−0.11]	0.29 ± 0.41 [0.22]*	0.37 ± 0.41 [0.36]*	0.57 ± 0.41 [0.71]*
Cognitive score, mean ± SD [median]	0.15 ± 0.29 [0.00]	0.42 ± 0.38 [0.32]*	0.47 ± 0.36 [0.32]*	0.73 ± 0.33 [0.86]*
Demographics				
Age, mean ± SD [median]	45.8 ± 12.6 [45.8]	42.4 ± 11.6 [42.8]	43.8 ± 14.7 [42.5]	52.7 ± 13.6 [53.3]*
Gender, n (%)				
Male	27 (55.1%)	44 (54.3%)	31 (70.5%)	31 (49.2%)
Female	22 (44.9%)	37 (45.7%)	13 (29.5%)	32 (50.8%)
Living arrangement, n (%)				
Independent	0 (0.0%)	12 (14.8%)	4 (9.1%)	15 (23.8%)
With caregiver	5 (10.2%)	12 (14.8%)	10 (22.7%)	12 (19.0%)
Group home	31 (63.3%)	30 (37.0%)*	19 (43.2%)	28 (44.4%)*
Group home and caregiver	7 (14.3%)	20 (24.7%)	5 (11.4%)	3 (4.8%)
Other living arrangement	6 (12.2%)	7 (8.6%)	6 (13.6%)	5 (7.9%)
Clinical characteristics				
Type of seizure, n (%)				
Bilateral seizures with motor symptoms	27 (55.1%)	32 (39.5%)	11 (25.0%)*	11 (17.5%)*
Focal seizures	36 (73.5%)	63 (77.8%)	26 (59.1%)	16 (25.4%)*
Epileptic spasms	4 (8.2%)	5 (6.2%)	2 (4.5%)	0 (0.0%)
Other manifestations of TSC, n (%)				
SEGA	16 (32.7%)	20 (24.7%)	14 (31.8%)	13 (20.6%)
rAML	41 (83.7%)	62 (76.5%)	31 (70.5%)	50 (79.4%)
TSC mutation, n (%)				
Had a test for gene mutations	48 (98.0%)	78 (96.3%)	42 (95.5%)	59 (93.7%)
*TSC1* mutation	5 (10.2%)	15 (18.5%)	10 (22.7%)	5 (7.9%)
*TSC2* mutation	27 (55.1%)	36 (44.4%)	20 (45.5%)	29 (46.0%)
Comorbidities, n (%)				
Skin abnormalities	36 (73.5%)	66 (81.5%)	33 (75.0%)	51 (81.0%)
Visual impairment	17 (34.7%)	27 (33.3%)	11 (25.0%)	17 (27.0%)
Skeletal disorder	34 (69.4%)	40 (49.4%)*	25 (56.8%)	35 (55.6%)
Cardiovascular problem	17 (34.7%)	41 (50.6%)	19 (43.2%)	27 (42.9%)
Sleeping disorder	8 (16.3%)	11 (13.6%)	1 (2.3%)*	6 (9.5%)
Level of daily functioning, n (%)				
Severe impairment	40 (81.6%)	53 (65.4%)*	28 (63.6%)	36 (57.1%)*
Mild or moderate impairment	5 (10.2%)	9 (11.1%)	3 (6.8%)	6 (9.5%)
No impairment	3 (6.1%)	19 (23.5%)*	13 (29.5%)*	20 (31.7%)*
Missing	1 (2.0%)	0 (0.0%)	0 (0.0%)	1 (1.6%)
Number of AED agents used, mean ± SD [median]	5.0 ± 2.7 [4.0]	5.6 ± 2.9 [5.0]	3.5 ± 1.8 [3.0]*	1.6 ± 1.2 [1.0]*
Healthcare resource utilization				
Patients with a visit during follow‐up time, n (%)				
Hospital	17 (34.7%)	40 (49.4%)	16 (36.4%)	9 (14.3%)*
Intensive care unit	3 (6.1%)	1 (1.2%)	1 (2.3%)	2 (3.2%)
Neurologist	33 (67.3%)	77 (95.1%)*	33 (75.0%)	27 (42.9%)*
Most common procedures during follow‐up time, n (%)				
EEG	14 (28.6%)	55 (67.9%)*	27 (61.4%)*	20 (31.7%)
MRI of the brain	6 (12.2%)	16 (19.8%)	10 (22.7%)	4 (6.3%)
CT scan of the brain	3 (6.1%)	20 (24.7%)*	4 (9.1%)	4 (6.3%)
Events				
Patients with an event during follow‐up time, n (%)				
Bilateral tonic‐clonic status epilepticus	11 (22.4%)	12 (14.8%)	5 (11.4%)	2 (3.2%)*
Nonconvulsive status epilepticus	0 (0.0%)	5 (6.2%)	0 (0.0%)	1 (1.6%)
Focal status epilepticus	0 (0.0%)	1 (1.2%)	1 (2.3%)	0 (0.0%)
Fractures	14 (28.6%)	11 (13.6%)*	2 (4.5%)*	0 (0.0%)
Injury or poisoning	4 (8.2%)	4 (4.9%)	4 (9.1%)	2 (3.2%)
Wounds	2 (4.1%)	2 (2.5%)	0 (0.0%)	2 (3.2%)
Behavioral impairment	0 (0.0%)	3 (3.7%)	0 (0.0%)	0 (0.0%)
Cognitive decline	0 (0.0%)	2 (2.5%)	0 (0.0%)	0 (0.0%)
Neurological insult	1 (2.0%)	0 (0.0%)	0 (0.0%)	0 (0.0%)

Abbreviations: AED, antiepileptic drug; rAML, renal angiomyolipoma; CT, computerized tomography; EEG, electroencephalography; EMG, electromyogram; HUI, Health Utility Index; IQ, intelligence quotient; MEG, magnetoencephalography; MRI, magnetic resonance imaging; PET, positron emission tomography; SD, standard deviation; SEGA, subependymal giant cell astrocytoma; SPECT, single‐photon emission computerized tomography; TSC, tuberous sclerosis complex; WADA, intracarotid sodium amobarbital test.

* Indicates *P*‐value < .05 compared to patients who are refractory 100% of the time. Chi‐square test was conducted for comparing categorical variables, and *t* test was conducted for comparing continuous variables.

Description and comparison of patients with TSC‐associated epilepsy stratified by impairment in daily functioning (N = 235) are presented in Table S2. Patients with impairment of daily functioning vs no impairment had lower overall HUI scores (mean 0.16 vs 0.75, *P* < .05) including also lower cognitive score (mean 0.30 vs 0.85, *P* < .05). In terms of clinical characteristics, a higher proportion of patients with impairment of daily functioning vs. no impairment had bilateral seizures with motor symptoms (37.8% vs 20.0%, *P* < .05), rAML (81.7% vs 63.6%, *P* < .05), and *TSC2* mutation (51.7% vs 30.9%, *P* < .05).

## 4 DISCUSSION

This real‐world study provided a unique opportunity for the evaluation of clinical factors associated with decrements in HRQoL in a large sample of patients with TSC, through a linkage of medical charts with patient/caregiver HRQoL self‐reports, including also considerable long‐term follow‐up for these patients. This study showed that epilepsy manifestations of TSC are associated with lower HRQoL compared to rAML manifestations only. Notably, the deficits in the cognition health dimension had the largest association with decrements in patients’ overall HRQoL. Additionally, in patients with TSC, older age, impairment of daily functioning, refractory epilepsy, and history of specific epilepsy‐related events were associated with a significantly lower HRQoL. The findings fill an important gap in the limited existing evidence on the impact of TSC‐associated epilepsy on HRQoL.

In this study, the prevalence of TSC‐associated epilepsy was 77.1%, which is slightly lower than in previous studies reporting an 83.5%‐93.5% lifetime prevalence of TSC‐associated epilepsy.^15^, ^36^, ^37^ Patients with TSC and epilepsy more commonly have multiple types of seizures, and more severe and refractory seizures, compared to patients with epilepsy due to other etiologies.^16^, ^38^, ^39^ Numerous prior studies have linked epileptic seizures to an increased risk of impairment of daily functioning.^40^, ^41^, ^42^ A recent European study of European and North American patients with TSC and epileptic seizures reported that health state utility values (HSUVs), index values representing HRQoL, incrementally decreased with the experience of more frequent and more severe seizures.^43^ This is also confirmed in the present study where the mean overall HUI score for patients with TSC and epilepsy (0.33) was considerably lower than in the general population (0.85) and was between the HUI score reported for patients with Alzheimer's disease (0.22) and rheumatoid arthritis (0.44).^28^, ^31^ Consistent with prior studies, the present study highlights the high clinical and economic burden of epilepsy in TSC, especially for patients who have seizures for prolonged periods of time.

Quality of life (QoL) is a broad concept closely linked to the overall well‐being of an individual within a society. When considering the results from this study, it is important to distinguish between QoL, HRQoL (an individual's or a group's perceived physical and mental health over time), and subjective happiness.^44^ In this study, overall HRQoL was low for patients with TSC‐associated epilepsy, especially for those with refractory epilepsy and severe impairment of daily functioning. However, most patients or caregivers who completed the HUI survey reported relatively high scores for the emotion (ie, happiness) dimension of the HUI, suggesting that most patients adjusted to their condition, limiting its emotional impact.

Since impairment of daily functioning, behavioral problems, and poor overall QoL are especially frequent in patients with longer duration of refractory epilepsy^45^, clinical guidelines emphasize the importance of frequent follow‐up and early adequate treatment of patients with TSC.^46^ Despite these efforts, up to 50% of patients have recurrent seizures with unsatisfactory response to pharmacologic and nonpharmacologic therapies.^46^ Currently, most pharmacological therapies for epilepsy, such as AEDs, primarily suppress seizures symptomatically, but lack disease‐modifying properties.^40^ In addition, AEDs are often associated with troublesome adverse effects (eg, behavioral problems, dizziness, sedation, insomnia, weight gain, and gastrointestinal upset).^46^, ^47^, ^48^, ^49^, ^50^ Surgical treatment may cure epilepsy or reduce seizure frequency and severity, but is limited to a small number of eligible patients with TSC due to the difficulty in identifying the epileptogenic zone.^40^

## 5 LIMITATIONS

Some of the limitations of this study are linked to its retrospective nature and the use of medical charts as a source to collect patient information. This includes inherent risks of incomplete or missing data within the medical record, records lacking specific patient information, difficulty in interpreting or verifying documented information, and variability in the quality of documentation among healthcare personnel. For example, some patients flagged as having refractory epilepsy in the database did not have any record in their medical charts for history of status epilepticus, fractures, injury, intoxication, wounds, behavioral impairment, cognitive decline, or neurological insult. While some of these patients may not have experienced such epilepsy‐related events in their lifetime, it is also possible that events occurring before the patient was referred to UMCU were not recorded. Other limitations specific to the current study may also apply. First, some patients who were invited to participate in the HUI‐3 assessment did not return the questionnaire; however, these patients were not different with respect to age, sex, living arrangement, TSC mutations, and most comorbidities, from patients who responded to the questionnaire. Second, for patients with severe impairment of daily functioning, the HUI‐3 questionnaire was filled out by a caregiver. Therefore, the overall HUI score for these patients may not be representative of their true HRQoL and may reflect the judgment of the caregiver. Third, the data and analyses are observational in nature, and therefore, the usual limitations regarding causal inference apply. Lastly, results may not be generalizable to settings outside of The Netherlands.

## 6 CONCLUSION

TSC patients with TSC‐associated epilepsy have considerable decrements in HRQoL and lower HRQoL than those with rAML only. In these patients, impairment in daily functioning showed the strongest association with decrements in patients’ overall HRQoL. The results from this study support the necessity of early and effective treatment interventions to reduce or control seizures, that may then help to preserve patients’ daily functioning, including cognitive functions, and improve overall HRQoL.

## CONFLICTS OF INTEREST

Dr Vergeer has received research grants from Analysis Group, Inc and Novartis Pharmaceuticals Corporation and declares employment at/ownership of Redgrasp. Dr de Ranitz‐Greven reports no disclosures. Dr Neary (currently Pfizer Inc) was an employee of Novartis Pharmaceuticals Corporation at the time the study was conducted. Dr Ionescu‐Ittu, Mr Emond, and Dr Duh are employees of Analysis Group, Inc, which received research grants from Novartis Pharmaceuticals Corporation for the conduct of this study. Dr Jansen reports no disclosures. Dr Zonnenberg received personal compensation from Novartis Pharmaceuticals Corporation. We confirm that we have read the Journal's position on issues involved in ethical publication and affirm that this report is consistent with those guidelines.

## Data Availability

Data and materials from this study cannot be publicly shared due to legal restrictions. Under the European Union data privacy laws, nonanonymized data cannot be used without the patient‐specific consent for each type of use. For any data requests, please contact the corresponding author.
