# Supplementary material for: Epilepsy, impaired functioning, and quality of life in patients with tuberous sclerosis complex
Source: Epilepsia Open. 2019 Oct 27;4(4):581–92. doi: 10.1002/epi4.12365 (PMC6885664; doi:10.1002/epi4.12365)
Supplement: Supplementary file 1 [file EPI4-4-0-s001.docx]

**Supplemental Table S1. Overall HUI-3 scores in different populations**

| **Age (years)** | **Population** | **Mean HUI-3** | **Reference** |
| --- | --- | --- | --- |
|  |  |  |  |
| 18+ | Patients with Alzheimer's disease | 0.22 | Neumann PJ, et al. med Decis Making 2000; 20(4) |
| 18+ | Patients with rheumatoid arthritis | 0.44 | Kaplan RM, et al. Med Care 2005; 43(1) |
| 21+ | Patients with chronic kidney disease stages 4 - 5 | 0.54-0.67 | Gorodetskaya I, et al. Kidney Int 2005; 68(6) |
| 5+ | Patients with Down syndrome | 0.55 | [Mok](#_ENREF_32) WK, et al. Health Qual Life Outcomes 2014; 12 |
| 18+ | Patients with difficult to control focal epilepsy investigated for epilepsy surgery | 0.56-0.61 | [Wiebe](#_ENREF_35) S, et al. J Neurol Neurosurg Psychiatry 2002;7392) |
| 12+ | Patients with both cancer and diabetes | 0.67 | Bowker SL, et al. Health and Quality of Life Outcomes 2006; 4(1) |
| 16+ | Patients with temporal lobe epilepsy, candidates for TLE surgery | 0.71 | [Wiebe](#_ENREF_35) S, et al. J Neurol Neurosurg Psychiatry 2002;7392) |
| 5+ | Survivors of brain tumors in childhood assessed at study entry (2+ yrs post-tx), and 5 and 10 years after | 0.77-0.88 | Duckworth J, et al. J Pediatr Hematol Oncol 2015; 37(5) |
| 18+ | Swedish general population | 0.85 | Neumann PJ, et al. med Decis Making 2000; 20(4) |

HUI: health utility index; TLE: temporal lobe epilepsy; tx: treatment; yrs: years

* Except for patients with TSC-rAML only, all other cohorts and subgroups investigated in this study appeared to have mean overall HUI scores below the reference score for the US in the general adult population and also below the HUI score previously reported for patients with cancer and diabetes or some forms of epilepsy without TSC.

**Supplemental Table S2. Characteristics of patients with TSC-epilepsy with and without impairment of daily functioning**

|  | **Patients with available information on impairment of daily functioning N = 235** | | | | |
| --- | --- | --- | --- | --- | --- |
|  | **Any impairment** | | **No impairment** | | **P-value** |
|  | **N = 180** | | **N = 55** | |  |
| Follow-up, years, mean ± SD [median] | 34.4 ± | 31.3 [12.6] | 30.7 ± | 27.7 [20.0] | 0.2026 |
| Time with refractory epilepsy, years, mean ± SD [median] | 19.3 ± | 23.4 [15.3] | 12.4 ± | 6.5 [13.5] | 0.0029* |
|  |  |  |  |  |  |
| **Quality of life** |  |  |  |  |  |
| HUI measurement, n (%) | 108 | (60.0%) | 37 | (67.3%) | 0.3315 |
| HUI score, mean ± SD [median] | 0.16 ± | 0.10 [0.38] | 0.75 ± | 0.78 [0.23] | <0.0001* |
| Cognitive score, mean ± SD [median] | 0.30 ± | 0.32 [0.34] | 0.85 ± | 0.96 [0.22] | <0.0001* |
|  |  |  |  |  |  |
| **Demographics** |  |  |  |  |  |
| Age, mean ± SD [median] | 12.0 ± | 2.5 [15.2] | 14.4 ± | 11.0 [15.2] | 0.2945 |
| Gender, n (%) |  |  |  |  |  |
| Male | 109 | (60.6%) | 24 | (43.6%) | 0.0267* |
| Female | 71 | (39.4%) | 31 | (56.4%) | 0.0267* |
| Living arrangement, n (%) |  |  |  |  |  |
| Independent | 2 | (1.1%) | 29 | (52.7%) | <0.0001* |
| With caregiver | 24 | (13.3%) | 14 | (25.5%) | 0.0326* |
| Group home | 105 | (58.3%) | 2 | (3.6%) | <0.0001* |
| Group home and caregiver | 31 | (17.2%) | 4 | (7.3%) | 0.0697 |
| Other living arrangement | 18 | (10.0%) | 6 | (10.9%) | 0.8455 |
|  |  |  |  |  |  |
| **Clinical characteristics** |  |  |  |  |  |
| Type of seizure, n (%) |  |  |  |  |  |
| Bilateral seizures with motor symptoms | 68 | (37.8%) | 11 | (20.0%) | 0.0146* |
| Focal seizures | 111 | (61.7%) | 29 | (52.7%) | 0.2371 |
| Epileptic spasms | 11 | (6.1%) | 0 | (0.0%) | - |
| Other manifestations of TSC, n (%) |  |  |  |  |  |
| SEGA | 48 | (26.7%) | 15 | (27.3%) | 0.9292 |
| rAML | 147 | (81.7%) | 35 | (63.6%) | 0.0051* |
| TSC mutation, n (%) |  |  |  |  |  |
| Had a test for gene mutations | 174 | (96.7%) | 51 | (92.7%) | 0.2052 |
| *TSC1* mutation | 23 | (12.8%) | 12 | (21.8%) | 0.0740 |
| *TSC2* mutation | 93 | (51.7%) | 17 | (30.9%) | 0.0115* |
| Comorbidities, n (%) |  |  |  |  |  |
| Skin abnormalities | 135 | (75.0%) | 50 | (90.9%) | 0.0116* |
| Visual impairment | 57 | (31.7%) | 15 | (27.3%) | 0.5361 |
| Skeletal disorder | 106 | (58.9%) | 26 | (47.3%) | 0.1286 |
| Cardiovascular problem | 82 | (45.6%) | 22 | (40.0%) | 0.4678 |
| Sleeping disorder | 22 | (12.2%) | 4 | (7.3%) | 0.3058 |
| Level of daily functioning, n (%) |  |  |  |  |  |
| Severe impairment | 157 | (87.2%) | 0 | (0.0%) | - |
| Mild or moderate impairment | 23 | (12.8%) | 0 | (0.0%) | - |
| No impairment | 0 | (0.0%) | 55 | (100.0%) | - |
| Number of AED agents used (anytime), mean ± SD [median] | 4.3 ± | 4.0 [2.8] | 3.2 ± | 3.0 [2.8] | 0.0121* |
|  |  |  |  |  |  |
| **Healthcare resource utilization** |  |  |  |  |  |
| Patients with a visit during follow-up time, n (%) |  |  |  |  |  |
| Hospital | 66 | (36.7%) | 15 | (27.3%) | 0.1995 |
| Intensive care unit | 7 | (3.9%) | 0 | (0.0%) | - |
| Neurologist | 131 | (72.8%) | 38 | (69.1%) | 0.5944 |
| Most common procedures during follow-up time, n (%) |  |  |  |  |  |
| EEG | 87 | (48.3%) | 29 | (52.7%) | 0.5684 |
| MRI of the brain | 25 | (13.9%) | 11 | (20.0%) | 0.2708 |
| CT scan of the brain | 24 | (13.3%) | 7 | (12.7%) | 0.9075 |
|  |  |  |  |  |  |
| **Events** |  |  |  |  |  |
| Patients with an event during follow-up time, n (%) |  |  |  |  |  |
| Bilateral tonic clonic status epilepticus | 29 | (16.1%) | 1 | (1.8%) | 0.0054* |
| Non-convulsive status epilepticus | 6 | (3.3%) | 0 | (0.0%) | 0.1702 |
| Focal status epilepticus | 1 | (0.6%) | 1 | (1.8%) | 0.3723 |
| Fractures | 23 | (12.8%) | 4 | (7.3%) | 0.2625 |
| Injury or poisoning | 12 | (6.7%) | 2 | (3.6%) | 0.4060 |
| Wounds | 6 | (3.3%) | 0 | (0.0%) | 0.1702 |
| Behavioral impairment | 2 | (1.1%) | 1 | (1.8%) | 0.6827 |
| Cognitive decline | 1 | (0.6%) | 1 | (1.8%) | 0.3723 |
| Neurological insult | 1 | (0.6%) | 0 | (0.0%) | 0.5796 |

AED: anti-epileptic drug; rAML: renal angiomyolipoma; CT: computerized tomography; EEG: electroencephalography; EMG: electromyogram; HUI: health utility index; IQ: intelligence quotient; MEG: magnetoencephalography; MRI: magnetic resonance imaging; PET: positron emission tomography; SD: standard deviation; SEGA: subependymal giant cell astrocytoma; SPECT: single-photon emission computerized tomography; TSC: tuberous sclerosis complex; WADA: intracarotid sodium amobarbital test.

* indicates p-value <0.05. Chi-square test was conducted for comparing categorical variables and t-test was conducted for comparing continuous variables.

**Supplemental Figure S1. Factors associated with HRQoL score in patients with TSC - univariate analysis**

CI: Confidence interval; HRQoL: Health-related quality of life; HUI: health utility index; rAML: renal angiomyolipoma; SEGA: subependymal giant cell astrocytoma; TSC: tuberous sclerosis complex.

* indicates p-value <0.05

**Note:**

^a^ Events include bilateral tonic clonic status epilepticus, non-convulsive status epilepticus, focal status epilepticus, other status epilepticus, unspecified status
